# Transradial Access for Radial-to-Peripheral Revascularization in Acute Limb Ischemia

**DOI:** 10.1016/j.jaccas.2026.108141

**Published:** 2026-05-12

**Authors:** Shadi Halabi, Ahmad Mohammad, Marwan Halabi, Serdar Farhan, Samer Abbas

**Affiliations:** a Department of Cardiology, Community Hospital, Powers Health, Munster, Indiana, USA; b Indiana University School of Medicine, Indianapolis, Indiana, USA; c Department of Cardiology, Lenox Hill Hospital, New York, New York, USA

**Keywords:** acute limb ischemia, peripheral intervention, superficial femoral artery, thrombectomy, transradial access

## Abstract

A 60-year-old woman with a history of peripheral artery disease and chronic kidney disease presented with Rutherford Class IIa acute limb ischemia of the right lower extremity. Transradial approach angiography revealed a 100% thrombotic occlusion of the superficial femoral artery. Successful lesion crossing, thrombectomy, and drug-coated balloon angioplasty were performed with restoration of TIMI flow grade 3 in a single session.

## Graphical Abstract

**Figure:**
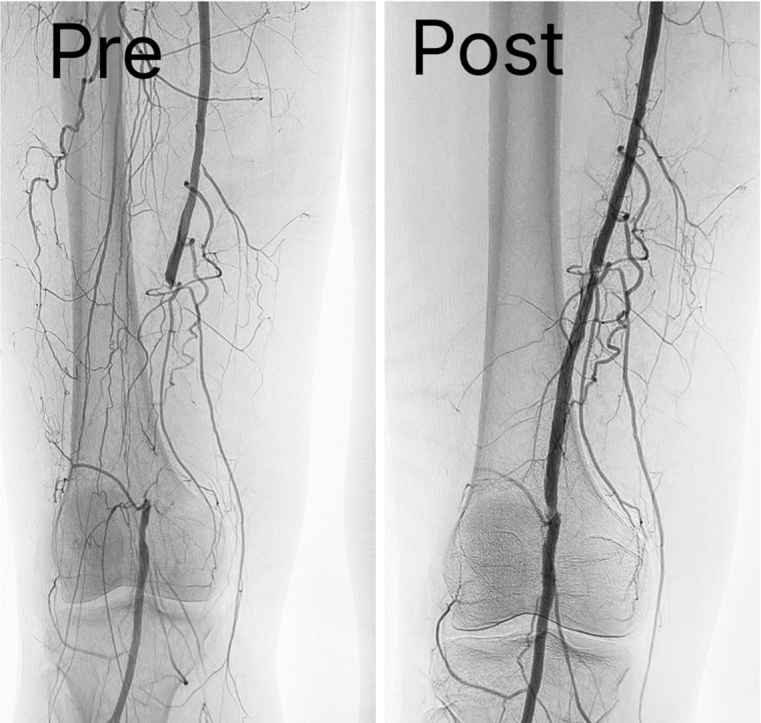

Acute limb ischemia (ALI) is a vascular emergency with high morbidity and mortality.^1^ The transfemoral approach (TFA) is the standard approach for treating ALI; however, it carries an increased risk of bleeding and access-site complications, especially in patients with obesity or those requiring aggressive anticoagulation. The transradial approach (TRA) offers a superior safety profile and earlier ambulation.^2^ TRA is increasingly used for treating patients with claudication.^3^

**Take-Home Message**

• The radial-to-peripheral strategy can be successfully used in selected patients presenting with acute limb ischemia.

To our knowledge, this case represents the first reported successful TRA revascularization for a patient presenting with ALI.

A 60-year-old woman presented with sudden-onset rest pain, coolness, and numbness in the right foot consistent with Rutherford class IIa ALI. The patient reported noncompliance with her prescribed rivaroxaban therapy. Physical examination of the right lower extremity revealed a cool limb with absent popliteal and pedal pulses, though signals were detectable by bedside Doppler.

Ultrasound-guided right radial artery access was obtained, and a 6-F sheath was placed. Angiography demonstrated a 100% thrombotic occlusion of the right superficial femoral artery using a 150-cm NAVICROSS Support Catheter (Terumo). The short radial sheath was subsequently exchanged for a 6-F 119-cm R2P (Radial-to-Peripheral) Destination Slender Guiding Sheath (Terumo). The lesion was successfully crossed with an 0.018-in GLIDEWIRE ADVANTAGE Guidewire (Terumo). Mechanical aspiration thrombectomy was performed using the 6-F 150-cm-shaft Lightning Bolt 6X aspiration catheter (Penumbra, Inc.). After successful thrombus removal, drug-coated balloon angioplasty was performed using a 200-cm-shaft Ranger Drug-Coated Balloon (Boston Scientific).

Final angiography showed the restoration of brisk flow to the foot with 3-vessel runoff (Figure 1). Immediate symptomatic relief was noted by the patient, and postprocedural examination confirmed warm skin temperature and palpable pedal pulses. Hemostasis was obtained at the radial site using a compression device, and the patient was discharged on the next hospital day.

**Figure 1:**
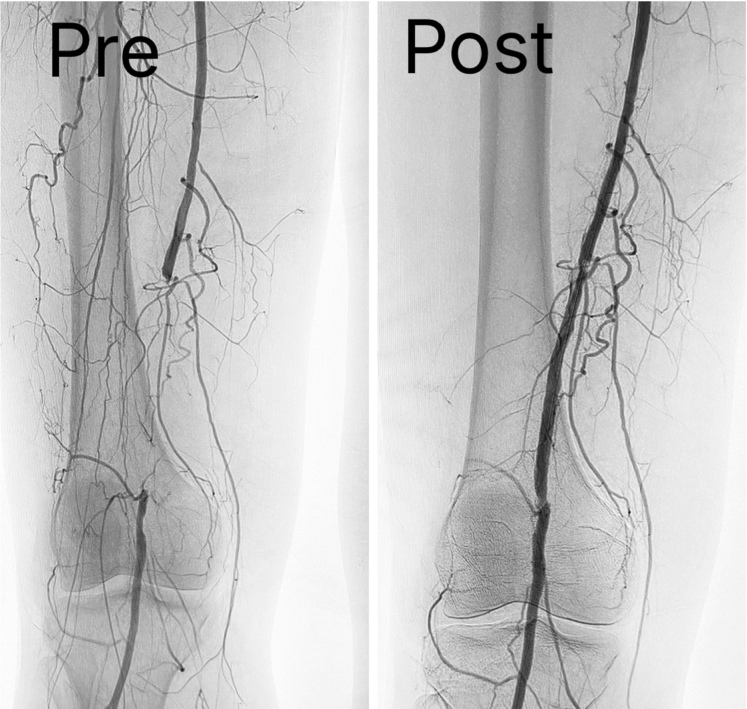
Transradial Mechanical Thrombectomy for Acute Limb Ischemia Pre: Right lower extremity angiography demonstrating 100% thrombotic occlusion of the distal superficial femoral artery and popliteal artery. Post: Final angiography after mechanical aspiration thrombectomy and drug-coated balloon angioplasty, showing complete restoration of flow (TIMI flow grade 3) with robust 3-vessel runoff down to the foot.

Despite that TRA is increasingly being used in coronary and peripheral artery disease intervention for claudication, its application in ALI remains sparsely reported. In this case, TRA was chosen over the traditional TFA because of the patient's high-risk profile: obesity, renal insufficiency (creatinine 2.08 mg/dL), and the requirement for therapeutic anticoagulation. The right radial artery was selected, given the patient's short stature and the proximal location of the superficial femoral artery occlusion.

Successful R2P interventions require careful case selection. Potential pitfalls include radial artery spasm due to longer sheaths, which can be mitigated by ensuring adequate radial artery size and using spasmolytic cocktails. Another limitation is the length of available devices, which may prevent reaching more distal lesions. Bailout strategies include conversion to TFA or using hybrid access approaches.

Recent technological advancements, including extended-length sheaths (119- to 149-cm R2P Destination Slender [Terumo]), long-shaft computer assisted vacuum thrombectomy (Lightning Bolt 6X, Penumbra Inc.), long-shaft balloons (200-cm shaft length; Ranger Drug-Coated Balloon [Boston Scientific] and Crosstella [Terumo]), and ultra-long guidewires (400- to 500-cm GLIDEWIRE ADVANTAGE [Terumo], F18 [Medtronic], and Viper [CSI]), now make TRA a safe and feasible alternative for lower-extremity interventions in patients with ALI.

## Funding Support and Author Disclosures

The authors have reported that they have no relationships relevant to the contents of this paper to disclose.
